# Algorithm for resolving discrepancies between claims for smoking cessation pharmacotherapies during pregnancy and smoking status in delivery records: The impact on estimates of utilisation

**DOI:** 10.1371/journal.pone.0202999

**Published:** 2018-08-30

**Authors:** Lucinda Roper, Duong Thuy Tran, Kristjana Einarsdóttir, David B. Preen, Alys Havard

**Affiliations:** 1 Centre for Big Data Research in Health (CBDRH), UNSW, Sydney, New South Wales, Australia; 2 Centre of Public Health Sciences and Unit for Nutrition Research, School of Health Sciences, University of Iceland, Reykjavik, Iceland; 3 Centre for Health Services Research, University of Western Australia, Perth, Western Australia, Australia; The University of Warwick, UNITED KINGDOM

## Abstract

**Background:**

The linkage of routine data collections are valuable for population-based evaluation of smoking cessation pharmacotherapy in pregnancy where little is known about the utilisation or safety of these pharmacotherapies antenatally. The use of routine data collections to study smoking cessation pharmacotherapy is limited by disparities among data sources. This study developed an algorithm to resolve disparity between the evidence of pharmacotherapy utilisation for smoking cessation and the recording of smoking in pregnancy, examined its face validity and assessed the implications on estimates of smoking cessation pharmacotherapy utilisation.

**Methods:**

Perinatal records (n = 1,098,203) of women who gave birth in the Australian States of Western Australia and New South Wales (2004–2012) were linked to hospital admissions and pharmaceutical dispensing data. An algorithm, based on dispensing information about the type of smoking therapy, timing and quantity of supply reclassified certain groups of women as smoking during pregnancy. Face validity of the algorithm was tested by examining the distribution of factors associated with inaccurate recording of smoking status among women that the algorithm classified as misreporting smoking in pregnancy. Rate of utilisation among smokers, according to original and reclassified smoking status, was measured, to demonstrate the utility of the algorithm.

**Results:**

Smoking cessation pharmacotherapy were dispensed to 2184 women during pregnancy, of those 1013 women were originally recorded as non-smoking as per perinatal and hospital data. Application of the algorithm reclassified 730 women as smoking during pregnancy. The algorithm satisfied the test of face validity—the expected demographic factors of marriage, private hospital delivery and higher socioeconomic status, were more common in women whom the algorithm identified as misreporting their smoking status. Application of the algorithm resulted in smoking cessation pharmacotherapy utilisation estimates ranging from 2.3–3.6% of all pregnancies.

**Conclusion:**

Researchers can use the algorithm presented herein to improve the identification of smoking among women who use cessation pharmacotherapies during pregnancy. Improved identification can improve the validity of safety analyses of smoking cessation pharmacotherapy—providing clinicians with valuable evidence to use when counselling women on the role of pharmacotherapy for smoking cessation during pregnancy.

## Introduction

Smoking in pregnancy is associated with poor maternal and fetal outcomes [1] yet is still relatively common [2, 3]. In 2013, it was estimated that between 11.3% and 15.0% of pregnant Australian women smoked [2, 3]. Less than half of these women quit spontaneously upon learning about their pregnancy, indicating that a large proportion are likely to require assistance to quit [4]. Psychosocial interventions have a modest impact on smoking rates during pregnancy [5]. The effectiveness of psychosocial interventions improves substantially when provided in combination with smoking cessation pharmacotherapies [6], however little is known about the safety of these pharmacotherapies in pregnancy [7–10], or the extent to which they are used by pregnant women [10–13]. Randomised controlled trials of varenicline or bupropion in pregnancy are unethical, due to limited evidence about their safety [7, 9, 10] and randomized control trials of nicotine replacement therapy (NRT) have faced difficulties with recruiting sufficient study participants [14, 15]. Research on utilisation and safety of smoking cessation pharmacotherapies in pregnancy is therefore largely limited to observational studies. Several observational studies suggested that during-pregnancy NRT use was not associated with increased risk of any major birth defects, but these studies reported inconsistent findings about the risk of preterm birth, stillbirth and low birth weight [16–19]. Evidence regarding the safety of bupropion is limited to a pilot trial and a record linkage study, which did not find increased risk of adverse outcomes [7, 20]. Evidence regarding the outcomes of varenicline use is limited to that from case series [10, 21].

Routinely-collected health data are a useful resource, because they allow for large population-based studies and reduce possible recall bias present in survey or questionnaire data. One of the concerns when using routinely collected data for pharmaco-epidemiological studies of smoking cessation medications is the validity and accuracy of smoking status recording. Estimates of utilisation will be altered by the extent to which the women who are smoking (and potential therapy users) are captured completely. Likewise, misclassification of smoking status can lead to inaccurate estimates of risk in safety analyses.

This study therefore focuses on how to optimise the ascertainment of smoking status from routinely-collected perinatal and hospital data from the Australian States of New South Wales (NSW) and Western Australia (WA). Previous work has indicated that perinatal data in Australia underestimate maternal smoking [22–24], therefore hospital diagnosis codes that indicate tobacco use in the last 28 days have been used to supplement the perinatal data, resulting in increased ascertainment of pregnant women who smoked by 9.6% [25]. Yet even when combined, these records under-ascertain smoking, compared to expected rates from the Australian Population Health Survey [25]. Previous studies in the United States of America [26, 27] and the United Kingdom [28, 29] also report under-ascertainment of smoking in pregnancy in population-based data.

This study was a part of the Smoking MUMS (Maternal Use of Medications and Safety) Study which investigates the utilisation and safety of smoking cessation pharmacotherapies in pregnancy [30]. We found that for nearly half of pregnant women who used a therapy both perinatal and hospital data did not indicate that they smoked during pregnancy. Such disparities could be due to the under-recording of smoking status or successful quitting before pregnancy with continued treatment for relapse prevention. This study’s first aim was to develop an algorithm, based on the pattern of pharmacotherapy supply during pregnancy, which can be applied to linked population health datasets to improve estimates of smoking in pregnancy among smoking cessation pharmacotherapy users. Given the absence of a gold standard data variable to ascertain smoking status in these women, the secondary aim was to examine the face validity comparing the distribution of factors known to be associated with the misclassification of smoking in pregnancy between women who were recorded as smoking in pregnancy and those whom the algorithm identified as having misreported their smoking status. Finally, the utility of the algorithm was demonstrated by demonstrating the extent to which it influenced estimates of smoking cessation pharmacotherapy utilisation during pregnancy.

## Methods

This is a cohort study design, based on data linked for the Smoking MUMS (Maternal Use of Medications and Safety] Study. The study comprised 1,098,203 pregnancies, belonging to 724,317 women, with a date of delivery between 1st January 2004 and 31st December 2012, to ensure that dispensing data were available for the entire gestation and 100-day pre-conception period.

### Data sources and linkages

Data sources, linkages and cleaning for the Smoking MUMS Study have been described elsewhere [30, 31]. In brief, this study linked perinatal, hospital and dispensing data from two Australian states. Firstly; the NSW Perinatal Data Collection and the WA Midwives Notification Scheme (collectively referred to as perinatal data). These contain delivery information (of all live and stillbirths of at least 20 weeks or over 400 grams birthweight), outcomes, demographic and medical information on the mother, including maternal smoking status. Secondly, this study used the NSW Admitted Patients Data Collection and the WA Hospital Morbidity Data Collection (collectively referred to as hospital data) which comprise all hospital separations (discharges, transfers and deaths), containing diagnoses (up to 55 in NSW and 23 in WA) which are coded according to the Australian Modification of the International Statistical Classification of Diseases and Related Problems, 10th revision (ICD-10-AM) [32] based on information in the medical record. The final dataset used was the Pharmaceutical Benefits Scheme data (referred to as dispensing data), which contain a record for every pharmaceutical product (product name, date of supply and prescription, quantity supplied and strength of therapeutic ingredient) for which a subsidy is paid under the Australian Commonwealth Government Pharmaceutical Benefits Scheme (PBS) [33]. Each medication was coded according to the Anatomical Therapeutic Chemical (ATC) classification system [34].

NRT patches, varenicline and bupropion are all available through the PBS if prescribed by a medical practitioner or nurse practitioner and dispensed in community pharmacies or private hospitals. Supply of these medicines to inpatients in public hospitals and over-the-counter purchases (applicable only to NRT, which is also available over-the-counter) are not PBS-subsidised and therefore not recorded in the dispensing data.

The linkage of perinatal data to hospital data was performed by the WA Data Linkage Branch and the NSW Centre for Health Record Linkage, while the Australian Institute of Health and Welfare linked perinatal to dispensing data. The linkages used probabilistic techniques and the best practice protocol for preserving individual privacy [35]. Quality assurance data show false-positive and false-negative rates of 0.3% and <0.1%, respectively, for NSW [36] and both are estimated to be 0.11% for WA [37, 38].

### Measures

#### Identifying smoking cessation pharmacotherapy use in pregnancy

All PBS records where any of smoking cessation therapies including bupropion (ATC code N07BA02), varenicline (N07BA03) and NRT patches (N07BA01) was dispensed to a woman at any point between 100 days prior to conception and date of delivery were identified. The minimum recommended course for non-pregnant adults is 7 weeks for bupropion, 12 weeks for varenicline and 8 weeks for NRT. Therefore, a full course required multiple dispensings, of those the earliest was referred to as the index supply. Days covered by each supply was calculated by dividing the dispensed quantity by recommended daily doses (twice daily for varenicline and bupropion [39, 40], and once daily for NRT) [41], then summed to derive the total days of supply to each woman. Women were defined as using smoking cessation pharmacotherapy during pregnancy if: i) any date of supply plus the days covered by that supply resulted in date that was after the date of conception; or if ii) the date of the index supply plus the total days supplied was beyond the date of conception.

A lookback period of 100 days prior to pregnancy was chosen to allow detection of a 12-week course of smoking cessation pharmacotherapy with a Medicines Possession Ratio (MPR) of at least 80%. Date of conception was calculated as the gestational age at birth (recorded in the perinatal data as number of completed weeks of gestation) subtracted from date of delivery plus 14 days [30].

#### Identifying recorded smoking status

**Fig 1:**
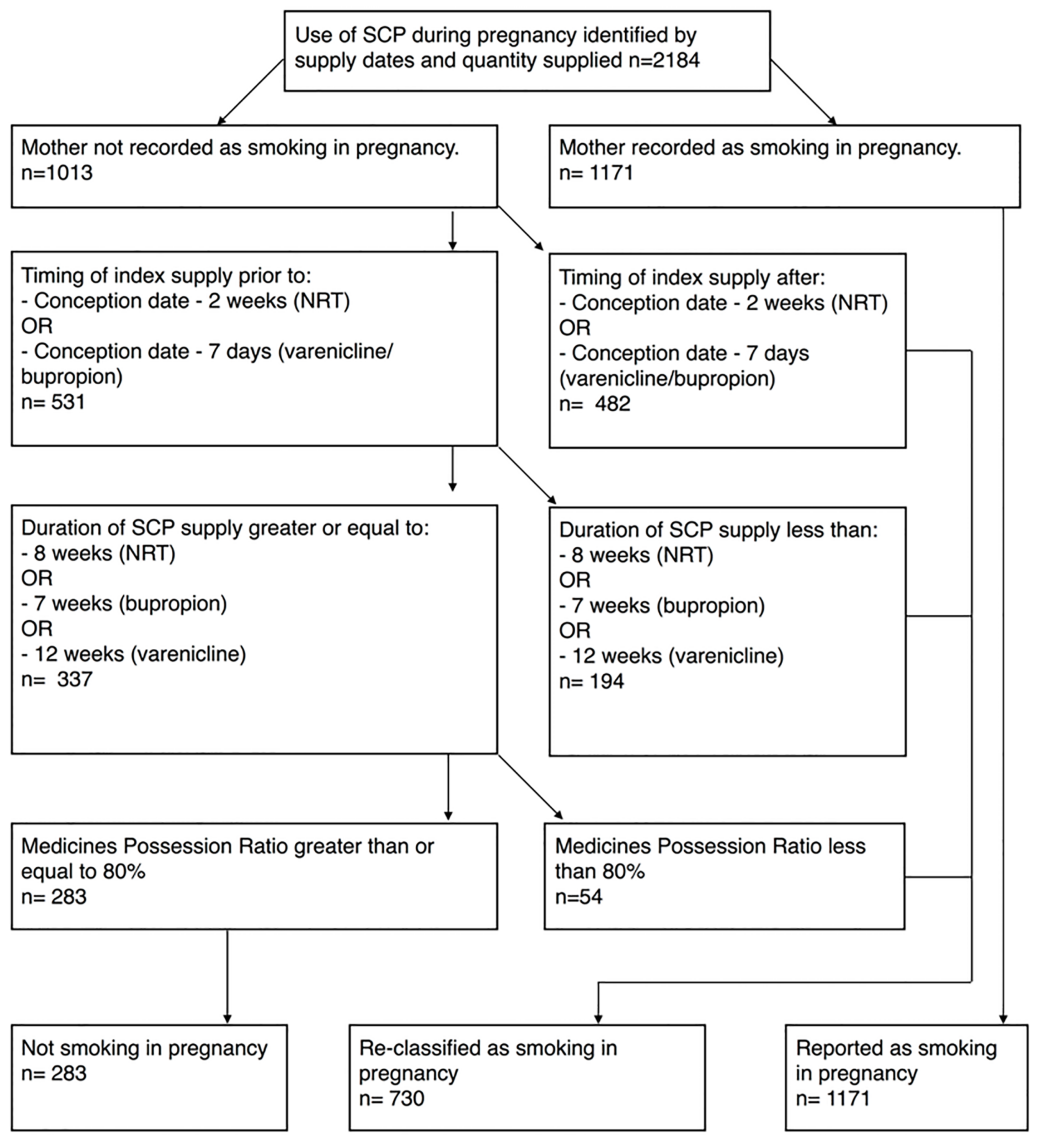
Application of the algorithm.

Perinatal data contained information that indicated whether women smoked tobacco at any time during pregnancy (prior to 2010 in WA, prior to 2011 in NSW) or during the first half and during the second half of pregnancy (since 2010 for WA and 2011 for NSW). In hospital data, the presence of an ICD-10-AM diagnosis code of Z72.0 code indicated current tobacco use, that is, the patient has smoked any amount of tobacco within the last 28 days [32]. In women who were identified as using a smoking cessation pharmacotherapy during pregnancy, the algorithm classified the mothers as smoking during pregnancy (Fig 1) if there was as a positive response on any of the smoking items in the perinatal data or there was a Z72.0 code recorded in any diagnosis field of the hospital separation record related to the delivery [25]. The remaining women (i.e. all negative responses in perinatal data and absence of the Z72 code in hospital data) were classified as mothers not recorded as smoking during pregnancy (Fig 1). If all fields contained a negative or no response, this was considered as smoking not reported.

#### Algorithm to reclassify smoking status among women recorded as not smoking

Based on a conservative approach which recognized that *potentially* successful quit attempt initiated pre-conception resulted in successful smoking cessation, the algorithm (Fig 1) used three criteria below to distinguish successful quit attempts from those that were unsuccessful in women recorded as not smoking. When all three criteria were met, the non-smoking status as recorded in perinatal and hospital data was maintained, whilst the remaining women were reclassified as smoking.

- *Timing of index supply*: The index supply must have been at least one week (in the case of varenicline or bupropion), or two weeks (in the case of NRT), prior to conception. NRT is recommended to be used for two weeks prior to quitting and varenicline and bupropion for one week before a pre-determined quit date [42].
- *Duration of therapy supply*: the total days of supplies were equal or longer than the minimum recommended course (8 weeks for NRT, 7 weeks for bupropion and 12 weeks for varenicline) [42]. Given the unknown safety of smoking cessation pharmacotherapy use in pregnancy, it is likely that women who are prescribed a smoking cessation pharmacotherapy for use during pregnancy are highly nicotine dependent, thus any use less than the minimum recommended course is unlikely to result in a successful cessation [19].
- *Medication adherence*: The Medicines Possession Ratio (MPR) was equal to or greater than 80% as per standard practice [43]. The MPR was calculated as:
  $$MPR=\frac{Total\;days\;of\;SCP\;supply}{(Date\;of\;the\;final\;supply+days\;supplied\;at\;final\;supply)-date\;of\;index\;supply}\times 100$$

Previous studies conducted among non-pregnant population [44–46] have shown strong relationship between therapy adherence and both short-term abstinence and long-term quit success. Whilst there are no studies of varenicline or bupropion adherence in pregnancy specifically, this relationship holds true for NRT use during pregnancy [47].

#### Examining the face validity of the algorithm

Descriptive statistics were generated, followed by unadjusted and adjusted analyses of factors associated with reclassification of smoking status, in order to test the face validity of the algorithm. Factors used for this test of face validity were identified from the literature regarding the accuracy of reporting of smoking in pregnancy. Under-reporting of smoking during pregnancy is associated with older maternal age (over 30 years), being married and having higher socioeconomic status [27, 28, 48–52]. Delivery in a private hospital is associated with less accurate recording of maternal smoking status in administrative data—it is unknown if this is due to maternal or hospital factors [25]. In this study, maternal age was obtained from perinatal data while marital status of NSW and WA mothers was obtained from the hospital data and the perinatal data, respectively. Variables relating to socioeconomic status of the mothers included Socio-Economic Index For Area (SEIFA) and concessional beneficiary status. The SEIFA Index of Relative Socio-economic Disadvantage scores for the mothers’ Statistical Local Area of residence (obtained from perinatal data) were grouped into quintiles [53]. The mother’s concessional beneficiary status indicated whether she received social welfare due to chronic illness, disability or low income. This variable was derived from the dispensing data record of smoking cessation pharmacotherapy supply, and categorized as general or concessional. The relationship between each of these factors and reclassification of smoking status was initially examined with crude odds ratios (ORs), followed by a single logistic regression in which all five factors were entered into a single adjusted model. Because several of these variables related to socioeconomic status, a check for collinearity was performed. Adding each variable to the model one at a time caused no change to the significance of the other variables in the model.

#### Examining the impact of the algorithm on smoking cessation pharmacotherapy utilisation

To examine the impact of the smoking status reclassification on estimates of smoking cessation pharmacotherapy utilisation, two sets of utilisation estimates for each therapy were generated. In both sets, the numerator comprised women who smoked during pregnancy and used smoking cessation pharmacotherapy, and the denominator was the number of women who smoked during pregnancy. The first set (lower bound estimate) was based on smoking status that was recorded in perinatal or hospital data while the second set (upper bound estimate) was based on smoking status reclassified by the algorithm. Utilisation was only estimated in pregnancies during which the particular smoking cessation pharmacotherapy was available. That is, bupropion utilisation was measured in all pregnancies in the cohort (PBS listed on the 1^st^ of February 2001); varenicline in pregnancies with conception date from 10^th^ of January 2008 (listing date), and NRT in pregnancies with a conception date from 1^st^ of February 2011 (listing date) to 31^st^ December 2012 (final delivery date included in cohort). The use of *any* smoking cessation pharmacotherapy was restricted to pregnancies with conception dates from the 1^st^ of February 2011 to a delivery date of the 31^st^ December 2012, during which all three smoking cessation pharmacotherapy were available.

All analyses were carried out in SAS version 9.4. Ethical approval for this study was granted by the NSW Population and Health Services Research Ethics Committee, the Australian Institute of Health and Welfare Ethics Committee, and the Department of Health WA Human Research Ethics Committee.

## Results

Amongst women who gave birth in NSW and WA between 1st of January 2004 and 31st December 2012, smoking cessation pharmacotherapy dispensing was identified for 2,184 pregnancies (Fig 1). These women received between one and three smoking cessation pharmacotherapy supplies (median one) during their pregnancy or the preceding 100 days. Mean maternal age was 29.2 years, and median parity was one.

In 1,013 (46.4%) of these pregnancies, smoking was not recorded in both perinatal and hospital data. In 6 of these 1013, this was due to a missing, as opposed to negative, response. Of these 1,013 pregnancies, 482 received the initial smoking cessation pharmacotherapy supply later than 2 weeks (NRT) or 1week (varenicline and bupropion) prior to conception, and were therefore re-classified as smoking in pregnancy. Of the remaining 531 with an initial script prior to the cut-off date, 194 received less than the minimum recommended course of smoking cessation pharmacotherapy and were reclassified as smoking in pregnancy. Of the remaining 337 who received at least the minimum dose, 54 had an MPR of less than 80% and were reclassified as smoking in pregnancy. This resulted in a total reclassification of 730 pregnancies. Therefore, after application of the algorithm there were 1,901 (87.0%) mothers who used a smoking therapy and smoked during pregnancy, and 38.4% of these had no smoking record in both the hospital and perinatal data.

### Factors associated with reclassification of smoking status

As seen in Table 1, there was a greater proportion of women who were married, over 30 years of age, delivered in private hospitals, lived in the highest SEIFA quintile and were PBS general patients among the women who were reclassified as smokers, compared to women recorded as smokers. After adjustment for all variables listed in Table 1, factors significantly associated with being reclassified included delivery in private hospitals (OR 1.59, 95% CI 1.2–2.5), PBS general patient status (OR 2.12, 95% CI 1.70–2.61) and being married (OR 1.50 95% CI 1.20–1.88). This indicates face validity of the algorithm, as women identified by the algorithm as misreporting their smoking status are be demographically similar to women identified as misreporters in past studies—higher socioeconomic status (non-concessional), married and delivered in a private hospital.

**Table 1:** Relationship between factors known to be associated with under-reporting smoking in pregnancy and reclassification of smoking status by the algorithm.

Factors associated with under-reporting	Recorded as smoking N = 1171 N (%)	Reclassified as smoking N = 730 N (%)	Reclassified smoking in pregnancy	
			Crude OR (95% CI)	Adjusted OR (95% CI)
**Maternal age**				
**≥30 years**	500 (42.7)	355 (48.6)	**1.27 (1.06–1.53)**	1.00 (0.82–1.22)
**<30 years**	671 (57.3)	375 (51.4)	1.00	1.00
**Marital Status**				
**Married**	703 (60.0)	551 (75.5)	**2.05 (1.67–2.52)**	**1.50 (1.20–1.88)**
**Not Married**	468 (40.0)	179 (24.5)	1.00	1.00
Socioeconomic Status (SES)*				
**1 (highest SES)**	126 (10.9)	152 (21.0)	**2.11 (1.53–2.91)**	1.39 (0.99–1.97)
**2**	283 (24.4)	200 (27.6)	1.24 (0.93–1.64)	1.03 (0.77–1.38)
**3**	300 (25.9)	152 (21.0)	0.89 (0.66–1.19)	0.84 (0.62–1.13)
**4**	227 (19.6)	93 (12.8)	0.72 (0.52–0.99)	0.69 (0.49–0.96)
**5**	224 (19.3)	128 (17.7)	1.00	1.00
**Hospital Type**†				
**Private**	69 (5.9)	105 (14.4)	**2.68 (1.95–3.69)**	**1.59 (1.2–2.5)**
**Public**	1099 (94.1)	623 (85.6)	1.00	1.00
**PBS Concessional Status**				
**General**	343 (29.3)	394 (54.0)	**2.83 (2.34–3.43)**	**2.12 (1.70–2.61)**
**Concessional**	828 (70.7)	336 (46.0)	1.00	1.00

*contain missing data;

^†^ all listed factors were entered simultaneously; bold text signifies statistically significant relationships

### Prevalence of smoking cessation pharmacotherapy utilisation

Table 2 presents the two sets of estimates of smoking cessation pharmacotherapy utilisation, stratified by type of smoking cessation pharmacotherapy and years that the smoking cessation pharmacotherapy was available on the PBS. When measured among the lower bound estimate prevalence of any smoking cessation pharmacotherapy use in pregnancy was 2.3%. In the upper bound estimate the prevalence increased to 3.6%, a relative increase of 56.5%. There was a relative increase in the prevalence of bupropion and varenicline utilization of 0.1% to 0.2% and 1.0% to 1.8%, respectively, and an increase in the estimate of NRT utilization of 30.8%.

**Table 2:** Smoking cessation pharmacotherapy utilisation rates.

	Smoking cessation pharmacotherapy utilisation among pregnancies where smoking was recorded (LOWER BOUND) n (%)	Smoking cessation pharmacotherapy utilisation among pregnancies where smoking was recorded *or* the algorithm reclassified as smoking in pregnancy (UPPER BOUND) n (%)
NRT in pregnancies from 1^st^ Feb 2011*	222 (1.3)	301 (1.7)
Bupropion in pregnancies from 1^st^ Jan 2004*	212 (0.1)	325 (0.2)
Varenicline in pregnancies from 10^th^ Jan 2008*	672 (1.0)	1199 (1.8)
Any smoking cessation pharmacotherapy in pregnancies from 1^st^ Feb 2011*	406 (2.3)	656 (3.6)

*Year stratification is based on the dates that each medication was available on the PBS

## Discussion

Among the 2184 pregnancies during which smoking cessation pharmacotherapy were dispensed, 53.6% had maternal smoking recorded in the perinatal or hospital data. After application of the algorithm, it was estimated that smoking occurred in 87.0% of pregnancies during which smoking cessation pharmacotherapy were dispensed. The finding that reclassified smokers were more likely to be married, have a higher socioeconomic status and to deliver in a private hospital, than women for whom smoking was recorded, is consistent with previous studies [25, 48–50, 52]. These findings confirm the face validity of the algorithm.

Of the total 1901 women found by the algorithm to smoke, 38.4% were not originally recorded as smoking in pregnancy. This is consistent with evidence that smoking is under-ascertained in Australian perinatal and hospital data. The magnitude of under-ascertainment is higher than that found in previous validation studies, but these have used medical records were used as the gold standard [22, 24]. The medical record is likely to underestimate smoking in pregnancy as there is no compulsory item on smoking status. It is also likely that there is genuinely greater under-ascertainment of smoking in pregnancy in the population using smoking cessation pharmacotherapy; women who have made attempts to *quit* smoking during pregnancy are more likely to misreport smoking to medical practitioners [49].

As smoking during pregnancy is under-ascertained in administrative data in a number of other countries, including the United States of America [26, 27] and the United Kingdom [28, 29], this method is likely to be of value internationally. The extent to which application of this algorithm will increase the identification of smoking in pregnancy, and therefore improve estimates of smoking cessation pharmacotherapy utilisation and safety, will vary according to the quality of the administrative data from which smoking in pregnancy is identified.

Whilst this study had access to dispensing data for all women with a perinatal data record, smoking cessation pharmacotherapy utilisation in pregnancy is typically only measured amongst women recorded as smoking in pregnancy in administrative data [12, 54]. The under-identification of smoking in pregnancy therefore has the potential to lead to inaccurate estimates of smoking cessation pharmacotherapy utilisation during pregnancy. As demonstrated in the current study, including undetected smokers in the cohort increases the prevalence estimate of smoking cessation pharmacotherapy utilisation by a relative 56.5%. It must be noted that this may be an overestimate, as undetected smokers who use smoking cessation pharmacotherapy were added to both the numerator and denominator of the estimate, whilst undetected smokers who do not use smoking cessation pharmacotherapy do not contribute to the denominator. Future studies of smoking cessation pharmacotherapy utilisation should therefore report both lower and upper bound estimates, to better contextualize the true prevalence.

The possibility of misclassification of smoking status amongst smoking cessation pharmacotherapy users has been acknowledged as a limitation in previous studies of smoking cessation pharmacotherapy safety [17, 18]. This study has indicated that 730 women smoking during pregnancy would be misclassified as non-smokers in smoking cessation pharmacotherapy safety analyses, if not identified by the algorithm. As a result, there would be inadequate control for the confounding effect of smoking on the outcomes examined, and inaccurate estimates of risk associated with smoking cessation pharmacotherapy would eventuate. The algorithm developed in the current study therefore has the potential to improve the accuracy of estimates of smoking cessation pharmacotherapy safety during pregnancy. By contributing to improvements in the quality of evidence available, this study may provide clinicians with the information to better counsel women about the risks and benefits of using smoking cessation pharmacotherapy during pregnancy.

Finally, this study is an exemplar of innovative use of multiple data sources to ascertain accurate information about lifestyle factors. This may be lacking from survey or questionnaire data regarding medications, environmental exposures or lifestyle factors, which iss frequently subject to recall bias.

## Limitations

Despite not being able to conduct a direct validation of the algorithm, the algorithm is based on expert content knowledge and satisfied a test of face validity. The algorithm is conservative in its reclassification of pregnant women’s smoking status, and we are therefore confident that the women reclassified as smokers were indeed smoking during pregnancy. In contrast, this conservative approach means that the women classified by the algorithm as not smoking in pregnancy may include some women who continued to smoke in pregnancy, but whose pattern of smoking cessation pharmacotherapy supply was suggestive of a possible successful quit attempt. This should be considered by future users of the algorithm.

Furthermore, dispensing data do not capture over-the counter medication purchases, and NRT is available both through the PBS (only patches are subsidised) and over-the-counter (all forms). Intermittent forms of NRT are considered safer than patches for use in pregnancy [4]. Yet there is a considerable price difference; in 2016 in Australia, subsidized NRT patches cost $114.90 or $12.40 (general and concessional patients, respectively) for 12 weeks, whereas 12 weeks of gum or lozenges cost from $336. Furthermore, NRT patches are popular amongst pregnant women, even when there is no price difference and they are well informed of the risks [55] [18]. Therefore, we expect that most women in Australia will choose to access the subsidized form of NRT. Nevertheless, any underestimate in NRT use may affect the algorithm in two ways. Firstly, women who only purchase over-the-counter NRT are not captured in the dispensing data, and would be classified as non-smoking cessation pharmacotherapy users with their smoking status remaining as recorded in the perinatal and hospital data. Secondly, some women who use both prescription and over-the-counter NRT may incorrectly remain classified as not smoking in pregnancy.

Finally, the algorithm could incorrectly reassign smoking status if any of the smoking cessation pharmacotherapy were used for a purpose other than smoking cessation. Bupropion is also used as an antidepressant in some countries, but is not licensed for use as an antidepressant in Australia. To be accessed through the PBS, the prescribing doctor must seek authority via phone [56], which ensures that bupropion is only prescribed for smoking cessation. Therefore, whilst off-label use is possible, it is unlikely to occur in Australia and thus does not present an issue.

## Conclusion

In many countries, administrative data under-ascertains smoking in pregnancy, which may compromise the accuracy of smoking cessation pharmacotherapy safety analyses. Using an algorithm designed to distinguish successful quit attempts from those that were likely to be unsuccessful, it was found that almost 40% of women using smoking cessation pharmacotherapy and smoking during pregnancy were not recorded as smoking in perinatal or hospital data. The algorithm for correcting smoking status has face validity, and enables the user to calculate an upper and lower bound estimate of smoking cessation pharmacotherapy utilisation, between which the true prevalence is likely to fall. Use of this algorithm in future Australian and international studies on smoking cessation pharmacotherapy use and safety has the potential to improve the quality of the evidence available to clinicians, who can, in turn, better support pregnant women who are attempting to cease smoking in pregnancy.
